# Penicillamine Neurotoxicity: An Hypothesis

**DOI:** 10.5402/2011/464572

**Published:** 2011-07-21

**Authors:** J. M. Walshe

**Affiliations:** Department of Medicine, Medical School, University of Cambridge, Cambridge CB2 2QQ, UK

## Abstract

Penicillamine, dimethyl cysteine, thiovaline, remains the drug of choice for the treatment of patience with Wilson disease. It is also of value in the treatment of cysteinuria and rheumatoid arthritis, it has also been suggested that it has value in the management of other rare diseases. It also has multiple toxicities. The majority of these can be explained as chemical toxicity, for instance its weak antipyridoxine action and its ability to interfere with lysyloxidea resulting in skin lesions. More important are its ability to induce immune reactions such as SLE, immune complex nephritis, the Ehlers Danlos syndrome and Goodpasture's syndrome. However the sudden increase in neurological signs which may occur in a small number of patients remains unexplained. The theory is proposed that this is due to lethal synthesis. In susceptible patients the–SH radical is liberated from penicillamine and will inhibit–SH dependent enzymes in the Krebs cycle leading to death in neurones. Other toxic metabolites may also be produced such as methyl mercaptan and ethyl mercaptan either of which could produce a similar metabolic block.

## 

After more than 50 years of experience penicillamine remains the most effective treatment for patients with Wilson disease for which there is ample evidence [1, 2]. However it also has a wide variety of toxicities, most of these can be explained in terms of chemical or immunological reactions. But one, the great increase in the neurological deficit remains unexplained. The present theories are unsatisfactory and this paper proposes a new hypothesis for the neurotoxicity of penicillamine which, if correct, may lead to the introduction of a predictive test which will be of great value in deciding whether or not this drug can be used as an initial therapy.

In 1948, when he established the role of copper in the pathogenesis of Wilson disease, Cumings [3] put possible therapy on a sound scientific basis by suggesting that the chelating agent BAL, British Antilewisite, might be beneficial in the management of this disease by removing the excess copper. This hypothesis was later supported by his own observations [4] and those of Denny Brown and Porter in Boston [5]. Later Denny Brown appeared to have abandoned the copper theory in favour of Uzman's hypothesis that the disease was due to an abnormality of peptide metabolism and that copper was merely deposited as a secondary phenomenon in dying neurones [6–8]. How Denny Brown was able to continue to propagate the use of BAL as an effective therapy in view of his theories on pathogenesis remains unclear [9]. However the peptide theory was finally disproved by Asatoor Milne and Walshe [10] when they showed that there were no abnormal peptides in the urine of Wilson disease patients and that the abnormal aminoaciduria could, in most cases, be corrected by treatment with penicillamine.

When, in 1956, I proposed that penicillamine might be of use as a chelating agent for copper removal [11] virtually nothing was known of its potential toxicity except that the L-isomer, when fed to ethanolamine deficient rats, caused loss of weight [12]. As the isomer liberated from penicillin is the the D isomer this did not seem relevant. I speculated on its potential toxicity and pointed out that as this aminoacid, in the D form, was present in the urines of all patients treated with penicillin it was therefore unlikely to have any immediate toxic effects and supported this by safely taking a gram myself. However the possibility of long-term toxicity had to be considered and I suggested “*dimethyl cysteine (penicillamine) might enter into the same metabolic pathways as cysteine and thereby cause a block. Occurring in the liver a metabolic block might lead to a conditioned cysteine deficiency and hepatic necrosis., in the skin it might cause alopecia as does selenium cystine. These points can be resolved only by long term study*”. Although a small number of patients have complained of excessive hair loss there have been no reports of total alopecia and hepatic necrosis also has not been reported. In practice, with increasing time of dosage and increasing number of patients treated, a wide spectrum of toxicities have been reported.

However, before any serious side effects were recorded came the report in 1963 and that penicillamine formed a mixed disulphide with cysteine and that this could be used for the treatment of another, unrelated metabolic disease, cystinuria [13] and Hartley and Walshe [14] were able to show that half the penicillamine excreted in urine was in this form. The first serious side effect to be reported was the nephrotic syndrome and this led directly to the development of trientine as an alternative orally active chelating agent [15]. As penicillamine toxicity became more of a problem the subject was dealt with in some detail in four symposia, 1968 [16], 1974, [17], 1977 [18], and 1981 [19], held on this drug. These can be best summarised in a diagram of 1985, (Figure 1). In the 1981 symposium [20] I pointed out that “*Penicillamine toxicity can be resolved into chemically and immunologically mediated reactions. Chemical toxicity is dose dependent and, if the drug is withdrawn in time, readily reversible.*” These reactions are also explicable in terms of the drugs known chemical reactions. For instance the skin lesions, cutis laxa, elastosis perforans, and epidermolysis can be explained in terms of the action of penicillamine on the copper-dependent enzyme lysyl oxidase which mediates collagen cross-linkage [21]. Similarly the ability of this drug to cause pyridoxine deficiency results from its ability to form a thiazolidine with the vitamin. Although, in fact, D-penicillamine has only a very weak action compared with that of the L isomer [22]. Much more serious are the immune reactions, such as SLE, the nephrotic syndrome, Goodpasture syndrome, and Ehlers Danlos syndrome and these all require immediate drug withdrawal.

A few toxicities remain unexplained, for example, the very rare mammary gigantism and the immediate and rapid increase in any or all of the neurological signs already present, whether they be dystonic, parkinsonian, or pseudosclerotic, once treatment has been started. The latter is serious and may leave the patient severely disabled or even prove fatal if treatment is not stopped immediately. Walshe and Yealland [23] found this in 11 of 137 patients with predominantly neurological signs. It has been suggested that this complication is due to the sudden release of ionic copper but this would seem unlikely as the copper so released is penicillamine bound and should be nontoxic. One possible mechanism has been suggested by Miki [24] “*However copper-penicillamine chelates are effective in catalyzing the oxidation of ghost membranes, which may be due to favourable alterations in the redox potential of copper. This acceleration of penicillamine may explain the initial aggravation of neurological symptoms often observed with its use.*” Another possible aggravating factor is the low level of urate found in the plasma following the renal leek of this metabolite [25] as this has antioxidant properties [26]. In support of this is the observation that patients with Parkinson's disease have a slower progression of their disease if the have higher serum and cerebrospinal fluid urate levels [27]. I have suggested an alternative explanation that in these patient there is a particularly unfavourable mutation, or combination of mutations. Since most patients are compound heterozygotes and as there are approximately 300 mutations on the affected gene, a P-type ATPase, it becomes difficult or almost impossible to prove, or disprove this hypothesis.

In view of the importance of this aspect treatment it would be advantageous to find a predictive test which could be used before embarking on any particular line of treatment. But before this can be achieved it is necessary to understand the underlying pathogenesis. I propose the following theory, this as example of what Rudolph Peters called lethal synthesis [28], that is a nontoxic compound, is changed, by enzyme action in the body, into a toxic one. Suppose that patients who react by a sudden neurological deterioration on starting treatment with penicillamine convert or break down the molecule into a powerful enzyme inhibitor. Normally penicillamine is either excreted unchanged or as the disulphide or as the mixed penicillamine-cysteine disulphide. Using radiolabelled penicillamine I found no evidence of other breakdown products in either the patient studied or in a normal control [29]. However if the SH group is removed from the molecule a free –SH radical or H2S, both highly toxic [30], might be released into the circulation and could prove powerful inhibitors of –SH dependent enzymes in the Krebs cycle, as also would other possible breakdown products, methyl mercaptan (CH3SH), and ethyl mercaptan (CH3CH3SH). Sudden inhibition of energy production by glucose oxidation would certainly prove disastrous to the CNS as neurons, especially those already damaged by copper, are heavily dependent on energy production from glucose. The removal of the SH alone would leave valine, an alternative name for penicillamine is thiovaline, a normally occurring amino acid, as an end product of this reaction. This leaves a possible test for this reaction The molecular weight of penicillamine is 149, that of valine 117. It follows that if a test dose of 1 gm of penicillamine be given to a patient and if all the SH were to be rapidly removed from the molecule this would leave 785 mg of valine in the circulation. Thus if an increase in plasma or urine valine levels was to be detected, this might provide a valuable test for identifying those patients particularly vulnerable to this form of penicillamine toxicity. An alternative approach would be to look for the appearance of the SH compounds, hypothesised above, in plasma. Another possible reaction has also been suggested [31] that “*If RS-radicals of penicillamine react with protein disulphide bridges with resultant formation of trisulphides, irreversible damage might be done. This may occur by a simple sulphur exchange mechanism RS + R′SSR – R + R′SSR′ or by a sequence of reactions trisulphide formations in proteins will exchange with the tertiary structure and in many cases would be harmful. Thus although penicillamine scavenges free radicals efficiently and repairs alkyl radicals rapidly, the thiyl radicals formed may not be harmless*”. If lethal synthesis explains this rare aspect of penicillamine neurotoxicity it does not necessarily explain the toxic reactions of other treatments, though it might be applicable to trientine it cannot be relevant to deterioration noted with zinc or tetrathiomolybdate where other mechanisms must be involved. 

##  Conflict of Interests

The author declares no conflict of interests. 

## Figures and Tables

**Figure 1:**
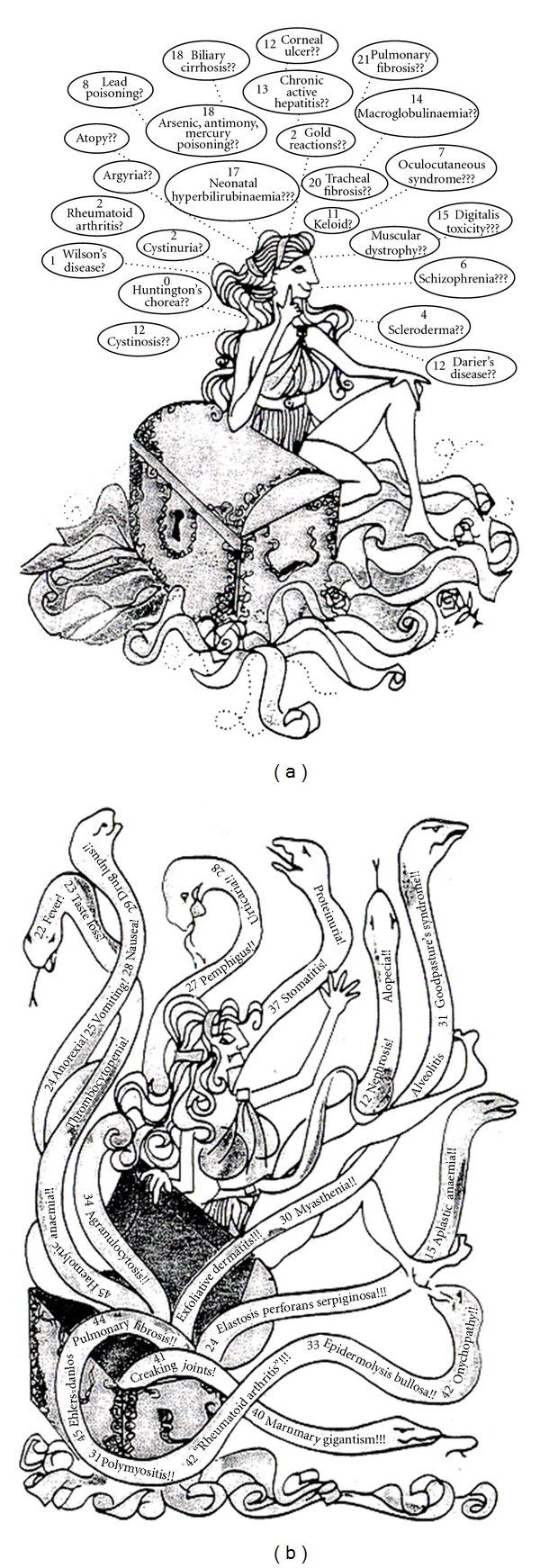
Penicillamine has many suggested beneficial actions as well as many toxicities, treasure trove or Pandora's box?
